# Global histone modification profiling reveals the epigenomic dynamics during malignant transformation in a four-stage breast cancer model

**DOI:** 10.1186/s13148-016-0201-x

**Published:** 2016-03-31

**Authors:** Quan-Yi Zhao, Pin-Ji Lei, Xiaoran Zhang, Jun-Yi Zheng, Hui-Yi Wang, Jiao Zhao, Yi-Ming Li, Mei Ye, Lianyun Li, Gang Wei, Min Wu

**Affiliations:** Department of Biochemistry and Molecular Biology, College of Life Sciences, Wuhan University, Wuhan, 430072 Hubei China; Division of Gastroenterology, Department of Geriatrics, Zhongnan Hospital, Wuhan University, Wuhan, Hubei 430072 China; CAS-MPG Partner Institute for Computational Biology, Shanghai Institutes for Biological Sciences, Chinese Academy of Sciences, Shanghai, 200031 China

**Keywords:** H3K9 methylation, Epigenomics, Breast cancer transformation, KDM3A, Transcription regulation

## Abstract

**Background:**

Epigenetic regulation has emerged to be the critical steps for tumorigenesis and metastasis. Multiple histone methyltransferase and demethylase have been implicated as tumor suppressors or oncogenes recently. But the key epigenomic events in cancer cell transformation still remain poorly understood.

**Methods:**

A breast cancer transformation model was established via stably expressing three oncogenes in primary breast epithelial cells. Chromatin immunoprecipitation followed by the next-generation sequencing of histone methylations was performed to determine epigenetic events during transformation. Western blot, quantitative RT-PCR, and immunostaining were used to determine gene expression in cells and tissues.

**Results:**

Histones H3K9me2 and me3, two repressive marks of transcription, decrease in in vitro breast cancer cell model and in vivo clinical tissues. A survey of enzymes related with H3K9 methylation indicated that KDM3A/JMJD1A, a demethylase for H3K9me1 and me2, gradually increases during cancer transformation and is elevated in patient tissues. KDM3A/JMJD1A deficiency impairs the growth of tumors in nude mice and transformed cell lines. Genome-wide ChIP-seq analysis reveals that the boundaries of decreased H3K9me2 large organized chromatin K9 modifications (LOCKs) are enriched with cancer-related genes, such as MYC and PAX3. Further studies show that KDM3A/JMJD1A directly binds to these oncogenes and regulates their transcription by removing H3K9me2 mark.

**Conclusions:**

Our study demonstrates reduction of histones H3K9 me2 and me3, and elevation of KDM3A/JMJD1A as important events for breast cancer, and illustrates the dynamic epigenomic mechanisms during breast cancer transformation.

**Electronic supplementary material:**

The online version of this article (doi:10.1186/s13148-016-0201-x) contains supplementary material, which is available to authorized users.

## Background

Recent advances in epigenetics and epigenomics have revealed that epigenetic abnormality is one of the critical causes for tumorigenesis [1–5]. DNA methylation inhibitors have already used for cancer treatment [6, 7]. Besides DNA methylation, abnormality of histone methylation is implicated in cancer as well. Early studies have shown that histone modification patterns can be used to predict tumor phenotypes and the risk of cancer recurrence [8–10]. Histone acetylation and deacetylation have been extensively studied and histone deacetylases (HDACs) are frequently reported to inhibit the expression of tumor suppressors [11]. Inhibitors of histone deacetylase (HDAC) are proved to be useful in clinical cancer treatment [11]. However, the relationship between cancer and other histone modifications, such as histone methylation, is still not conclusive.

Histone methylation usually occurs on lysines and arginines, and each site has three different forms [12, 13]. In comparison with acetylation, histone methylation and demethylation have more complicated regulatory steps, making them promising drug targets [1, 14]. Recently, several well-studied histone methyltransferases emerged to be key regulators in multiple cancer types. For example, enhancer of zeste 2 polycomb repressive complex 2 subunit (EZH2), the major enzyme for H3K27me3, was demonstrated as an oncogene in prostate cancer [15]. Its mutation has also been frequently found in lymphoma [16, 17]. An enzymatic inhibitor of EZH2 was shown to inhibit lymphoma cell proliferation in a mouse model [18]. SET domain containing 2 (SETD2) is the major H3K36me3 methyltransferase in mammalian cells and frequently mutated in clear cell kidney carcinoma, acute leukemia, gliomas, and other cancers [2, 19–22]. H3K36me3 catalyzed by SETD2 is required for genome stability and DNA repair process after damage [23–25]. Myeloid/lymphoid or mixed-lineage leukemia 1-4 (MLL1-4), the methyltransferases for H3K4, was found to be mutated in multiple types of cancers [2, 4]. On the other hand, demethylation is another critical aspect for the dynamic regulation of histone methylation. Clinical studies from different groups found that the demethylases of H3K9me2 and me3, such as lysine (K)-specific demethylase 3A (KDM3A, also known as JMJD1A or JHDM2A) and lysine (K)-specific demethylase 4A/B/C (KDM4A/B/C), are highly expressed in cancer tissues and regulate tumorigenesis [26–29]. Moreover, KDM4A was reported to induce site-specific copy gain and DNA re-replication and promote cellular transformation by inhibiting p53 signaling [26, 30]. A histone H3K4 demethylase, lysine (K)-specific demethylase 5A (KDM5A), is involved in the cancer cell drug tolerance [31]. All these information suggests that histone methylation is critical in the genesis and development of multiple cancer types. However, the molecular mechanisms of these enzymes in tumorigenesis still remain elusive.

The early diagnosis and treatment are the most effective ways to cure cancer. But we are still lack of good markers and drugs for precision medicine. During transformation, the epigenetic programs in cells change dramatically, helping them to survive and gain growth advantage [1, 3]. Along with the development of functional genomics, some studies have investigated the dynamic changes during differentiation [32], but the epigenetic dynamics at the genome-wide scale during transformation are still not known. However, due to the individual variation, cell heterogeneity, and limited sample size, patient tissues are difficult for mechanistic studies. Thus, a transformation model with clean background and high reproducibility is required. More than a decade ago, a cell-based model was established to mimic transformation from human primary cells to tumor cells [33, 34]. With the introduction of three genes, large T antigen, telomerase reverse transcriptase (*TERT*), and Harvey rat sarcoma viral oncogene homolog v12 mutant (*RAS* (*V12*)), the engineered primary cell will extend life span, become immortalized, and finally gain tumorigenic capacity, which is considered to represent different stages of tumor cell transformation [33, 34]. Considering the difficulties in studying human cancers, the in vitro model still serves as one of the best platforms to study the molecular mechanisms of tumor transformation. Recently, profiling of global gene expression in the above model has provided valuable information for tumor transformation study and suggested a link between malignant transformation to de-differentiation [35]. But due to the difficulty in making the model, no epigenetic study was reported.

In this study, we took advantage of the in vitro tumor transformation model and made a series of transformed cell lines starting from human primary mammary cell (HMC). By combining biochemical and epigenomic approaches, we demonstrate that histones H3K9me2 and H3K9me3 decrease during breast cancer transformation and contribute to the process with different mechanisms. Furthermore, we identified that KDM3A/JMJD1A, an H3K9me2 demethylase, is responsible for the H3K9me2 reduction and critical for breast tumor transformation.

## Results

### Gene expression profile of tumor transformation model mimics clinical samples

To study the underlying mechanisms of breast cancer transformation, we utilized an established cell-based transformation model [34, 35]. Large T antigen, *TERT*, and *RAS* (*V12*) were stably expressed in human primary mammary cell respectively via retroviral infection. Four cell lines were generated for following studies, namely HMC-p6 (human primary mammary cell, passage 6), HMC-L (HMC with large T stable expression), HMC-LT (HMC with large T and *TERT* stable expression), and HMC-LTR (HMC with large T, *TERT*, and *HRAS* (*V12*) stable expression) (Additional file 1: Figure S1A, B). HMC-p6 and HMC-LT can be passaged for 1 and 2 months, respectively, and HMC-LT is immortalized (Additional file 1: Figure S1C). Only HMC-LTR can form colonies in soft agar and grow into tumors in nude mice (Additional file 1: Figure S1D). These observations are in full accordance with previously reported results [34].

**Fig. 1:**
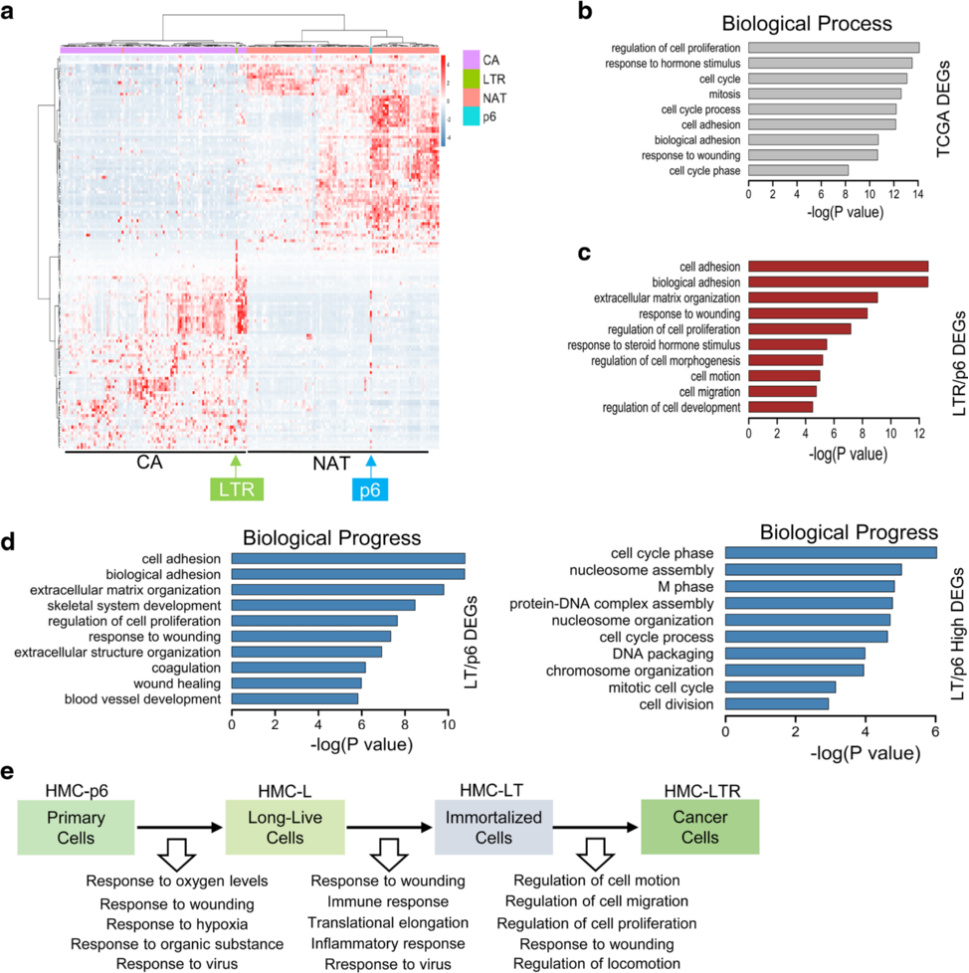
Tumor cell line derived from primary mammary cell mimics clinical breast cancer tissues. **a** HMC-p6, HMC-LTR, TCGA 100-paired breast cancer, and normal tissues are clustered by the expression of the overlapped differentially expressed genes (DEGs). Hierarchical cluster heatmap is shown using Ward method. **b**, **c** Biological process (BP) enrichment analysis of TCGA and LTR total DEGs. GO analysis was performed with DAVID, and items were ordered by *P* value. **d** BP enrichment analysis of LT/p6 (*left*) and LT/p6 up-regulated (*right*) DEGs. **e** A sketch map of biological processes changed during each step of cell transformation according to their gene expression profiles

**Fig. 2:**
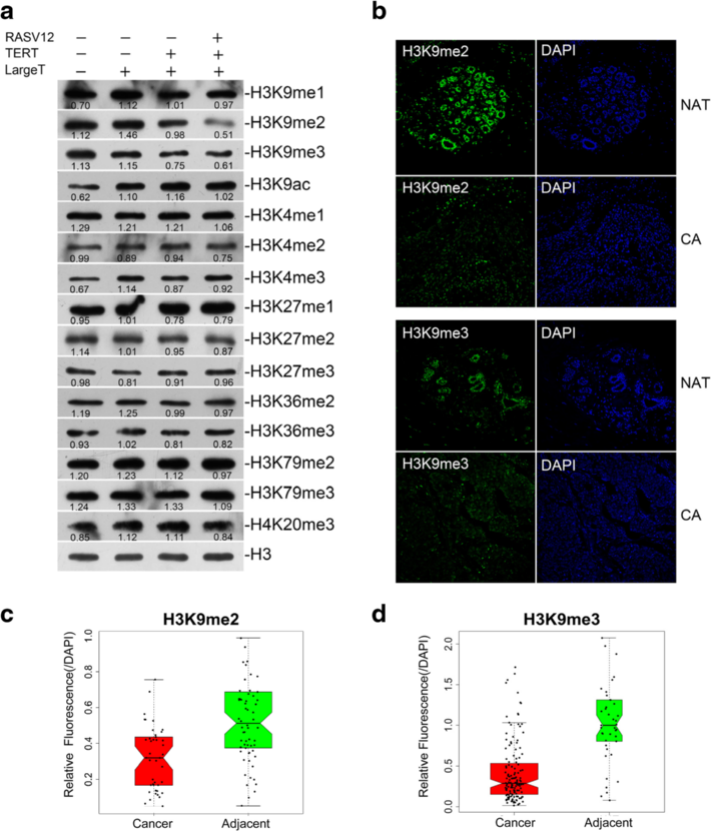
Reduction in H3K9me2 and me3 levels in transformed cell lines and breast cancer tissue. **a** Profile of histone modifications in transformed cell lines by Western blotting. The relative level of each modification vs histone H3 was measured with ImageJ and labelled below the corresponding band. H3K9me2 and me3 levels decrease along with transformation, coupled with H3K9ac increase. **b** Decreased H3K9me2 and me3 in breast cancer tissues. Commercially purchased paraffin tissue chips including normal adjacent tissue (NAT) and breast cancer (CA) were stained with fluorescent antibodies. **c**, **d** *Box plots* displaying fluorescence densities of H3K9 me2/me3 in cancer and normal tissues. Fluorescence densities were measured by Olympus FV1000. *Dots* represent the ratio of H3K9 methylation/DAPI in each sample. *P* value <0.05. *P* values were calculated by Wilcoxon rank sum test

Based on their ability in proliferation and tumorigenicity, the four cell lines are considered to represent different transformation stages. To further confirm the validity of the tumor cell model, we clustered the transformed cell lines with clinical samples based on their expression profiles of differentially expressed genes (DEGs). Gene expression profiles of 100 cases of paired breast cancer and normal tissues were downloaded from the Cancer Genome Atlas (TCGA), and the DEGs (twofolds) were analyzed according to the pipeline described in experimental procedure (Additional file 1: Figure S2A and Additional file 2: Table S6). These DEGs were then compared with those (threefolds) identified from transformed HMCs (Additional file 1: Figure S2A and Additional file 2: Tables S1–S5), and the expression patterns of resulting 338 genes were used for clustering (Fig. 2a, Additional file 1: Figure S2B, C and Additional file 2: Table S7). Cluster analysis grouped HMC-LTR with breast cancer tissues and HMC with adjacent tissues (Fig. 1a), suggesting the transformed LTR cell line may partially mimic some of the clinical samples. HMC-p6, -L, and -LT are grouped together, while HMC-LTR is separate, which may indicate the big difference of tumor cell line with others (Additional file 1: Figure S2B). We further used the DEGs of HMC-p6 and -LTR to cluster the TCGA tissues and successfully grouped them into normal and cancer groups (Additional file 1: Figure S2C).

Furthermore, gene ontology analysis showed that the tumor transformation model shares similarities with clinical samples (Fig. 1b, c, Additional file 1: Figure S2D, F). The DEGs of HMC-LT and -LTR in comparison with HMC-p6 were enriched mostly in cell cycle regulation and extracellular environment (Fig. 1c, d), which were both enriched in clinical samples (Fig. 1b). Then, we analyzed the gene expression profiles of all the four cell lines. We found that the most significant enriched processes are the following: (1) response to oxygen and hypoxia for p6 to HMC-L, (2) response to wounding, immune, and inflammatory response for HMC-L to HMC-LT, (3) regulation of cell motility, migration, and proliferation for HMC-LT to HMC-LTR (Fig. 1e and Additional file 2: Tables S1–S5). All these processes are frequently activated in tumors. Our analysis provides important information of precancerous transformation.

When we initiated our study, we randomly selected some datasets from the TCGA database. The analysis did not distinguish HMC-p6 and -LTR as good as that in Fig. 1a, and HMC cells clustered much better with adjacent normal tissues in the new analysis (Fig. 1a, Additional file 1: Figure S2G, H). The difference between the two analysis is as follows: (1) More tissues were used in the new analysis (100 paired tissues) compared with the early one (70 cancer and 12 normal tissues); (2) paired tissues are more useful than random samples because less individual difference leads to less disturbance; (3) more strict conditions were used for DEGs among tissues (threefolds compared with twofolds, Additional file 1: Figure S2A, G). These experiences might be useful to the other similar analysis with online large-scale sequencing data.

### Profiling of histone modifications during transformation

In order to systematically characterize epigenetic changes during transformation, we profiled the four cell lines with available commercial antibodies for histone modifications. Interestingly, we observed a gradual reduction of H3K9me2 and me3 along with transformation (Fig. 2a). On the contrary, H3K9 acetylation showed a gradual elevation (Fig. 2a). No obvious changes were observed for other modifications (Fig. 2a). Next, we studied if our discovery in in vitro cell model is also the same in clinical patient tissues. We performed immunostaining in commercial breast cancer tissue arrays and observed a strong reduction in cancer tissues for both methylations (Fig. 2b). In total, we analyzed 140 cancer and 40 adjacent samples for H3K9me3 and 42 cancer and 66 adjacent samples for H3K9me2. Statistical analyses of fluorescence densities representing H3K9 me2 and me3 show obvious lower methylation levels in cancer tissues (Fig. 2c, d).

### The altered transcription programs of cancer-related genes regulated by H3K9me2

**Fig. 3:**
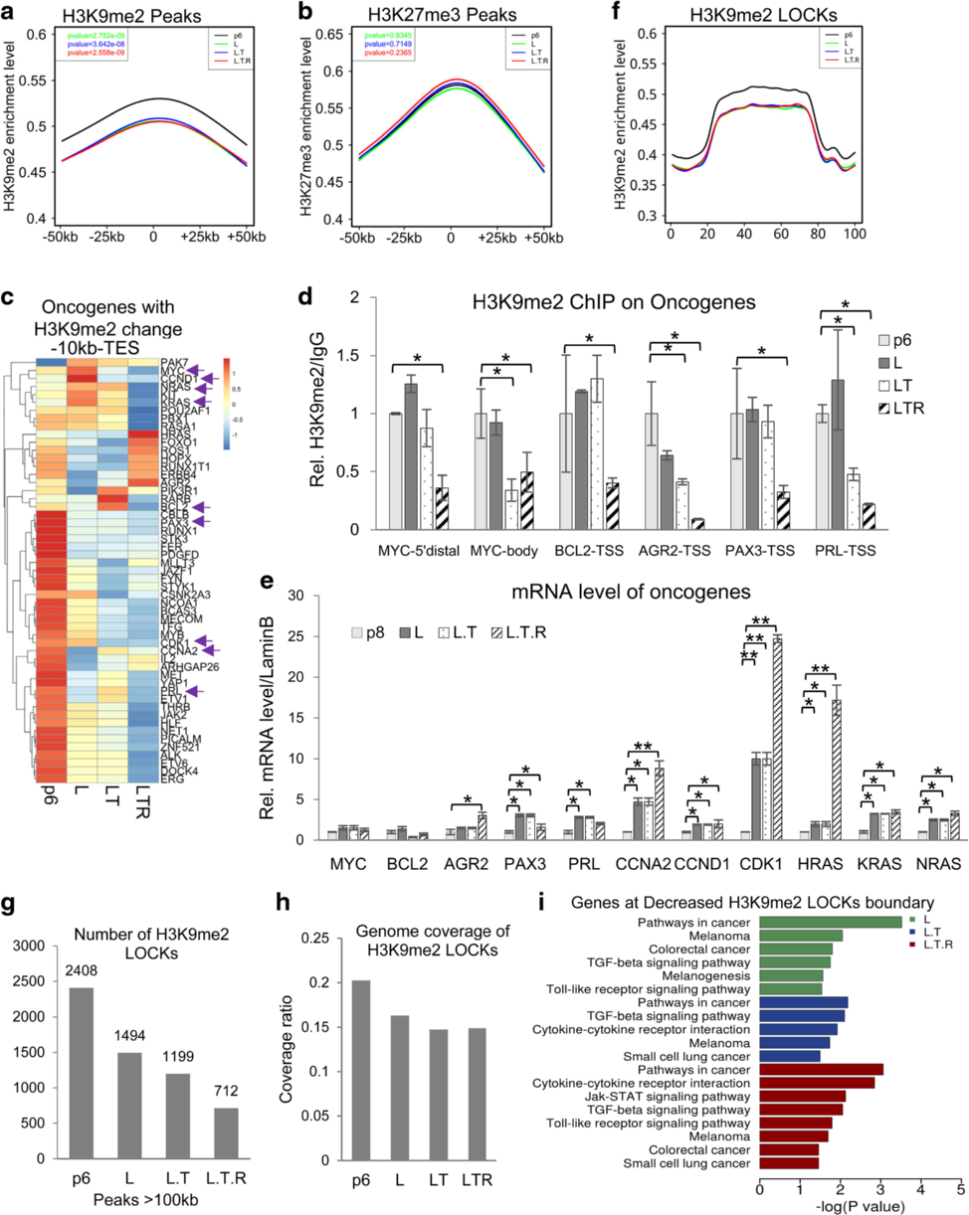
Decreased H3K9me2 alters the transcription program of cancer-related genes at the boundaries of LOCKs. **a**, **b** Average enrichment levels of H3K9me2 (**a**) and H3K27me3 (**b**) around detected peaks. **c** The heatmap shows the dynamic H3K9me2 levels on the oncogenes with H3K9me2 reduction. The reads of all H3K9me2 peaks from −10 kb of TSS to TES on each gene were used. The genes verified later by ChIP-PCR and RT-PCR are labelled by *arrows*. **d** H3K9me2 levels on several oncogenes were confirmed by ChIP-PCR. **e** The mRNA expression level of oncogenes with decreased H3K9me2 in transformed cell lines. **f** The average levels of H3K9me2 LOCKs in the four cell lines. Peaks with length >100 kb were defined as LOCKs. All LOCKs in the four cell lines were extended down- and upstream for 50 kb, and their average H3K9me2 enrichment levels were calculated. **g**, **h** The number and average coverage of H3K9me2 LOCK number in four cell lines. **i** KEGG pathway analysis of genes located at the boundaries of LOCKs with decreased H3K9me2

To further confirm our discovery, we performed ChIP-seq studies in the four cell lines of H3K9me2, H3K4me3, and H3K27me3. The data suggested H3K9me2 reduced significantly in HMC-L, -LT, and -LTR; H3K27me3 had no significant change (Fig. 3a, b). H3K4me3 enrichment seemed reduced also in transformed cell lines but not significant enough (Additional file 1: Figure S3G). However, we did not see a gradual change by high throughput sequencing among the later three cell lines, which is different from western. The reason might be that two methods used different standards for normalization, histone H3 for western and input for ChIP-seq. Nevertheless, our results indicate that histone H3K9me2 decreased significantly during transformation.

We then analyzed the dynamic changes of H3K9me2 distribution during tumor transformation. A slight increase of H3K9me2 peaks on distal intergenic regions during transformation was observed (Additional file 1: Figure S3A–C). The length of all H3K9me2 peaks and decreased peaks reduced modestly during the process, while the length of increased peaks remained unchanged (Additional file 1: Figure S3D, E). The biological meanings for these changes still required more studies. Interestingly, we also found that the length of H3K27me3 peaks decreased along with transformation (Additional file 1: Figure S3F), though its total enrichment did not change significantly (Fig. 3b). Various mutations and abnormal expression of EZH2, the major enzyme catalyzing H3K27me3, are associated with cancers [15, 17, 18]. Our discovery provides a clue to the study how H3K27me3 dysregulation is involved in breast cancer transformation.

We noticed that decreased H3K9me2 peaks were mainly located on gene bodies (Additional file 1: Figure S3C), therefore, the relationship between gene expression and H3K9me2 during transformation was investigated. Considering the importance of promoters for gene expression, the total reads of H3K9me2 peaks from −10 kb before TTS (usually considered as proximal promoter region) to TES was calculated. We found that H3K9me2 dynamically changed on many oncogenes (Fig. 3c). We then confirmed the change of histone modifications on some of them (Fig. 3d and Additional file 1: Figure S4A). We further analyzed their messenger RNA (mRNA) levels and confirmed with quantitative PCR that many genes exhibited correlated expression (Fig. 3e and Additional file 1: Figure S4B–C). Meanwhile, we found that a few genes did not show the expected elevated expression, such as MYC and BCL2 (Fig. 3e). H3K9me2 is generally believed to be a transcription repression mark and its reduction is required for gene activation. However, H3K9me2 reduction on genes may not directly lead to transcription alteration, and upstream signals sometimes are required.

We also analyzed H3K4me3 and H3K27me3 on genes with decreased H3K9me2. Surprisingly, the average levels of these marks on the above genes are all lower than that in primary cell (Additional file 1: Figure S4D, E). Recently, several groups reported that synergistic decrease of histone H3K4, K9, and K27 methylation is associated with the increase of DNA methylation [36, 37]. It is possible that some genes with H3K9me2 decrease may be also involved in DNA methylation changes.

### Boundaries of H3K9me2 LOCKs are enriched with cancer-related genes

H3K9me2 often modifies broad regions on chromatin, which have been previously named as large organized chromatin K9 modifications (LOCKs) [38]. The H3K9me2 on LOCKs has a similar pattern as the total peaks, high in HMC and low in the other three cell lines (Fig. 3f). The total numbers and genome coverages of H3K9me2 LOCKs decreased during transformation (Fig. 3g, h). We analyzed the genes overlapped with all decreased H3K9me2 LOCKs but did not find any relationships with cancer. We speculated that change of a broad chromatin region may start from its boundaries, so we analyzed the genes located on the boundaries of the decreased LOCKs. Surprisingly, we found that the genes located in these boundaries are enriched with cancer-related pathways (Fig. 3i and Additional file 2: Tables S8–S10). This suggests that the localization of genes in H3K9me2 LOCKs is related with cellular functional changes and unlocking the oncogenes at the boundaries facilitates the transformation process. The genes at decreased H3K9me2 LOCK boundaries in three transformed cells are very similar, including *BTG1*, *MYC*, *PIK3R1*, and *WNT5A* for oncogenes and *CDKN2A*, *CDKN2B*, *FH*, *INTS6*, *LINC00032*, *MITF*, *PLK2*, and *WNT5A* for tumor suppressors (Additional file 2: Table S11). *MYC* is the well-known oncogene in breast cancer and many other cancer types [39]. All the other genes may also play important roles in breast tumor transformation.

### High expression of H3K9 demethylase KDM3A/JMJD1A in breast cancer cell lines

**Fig. 4:**
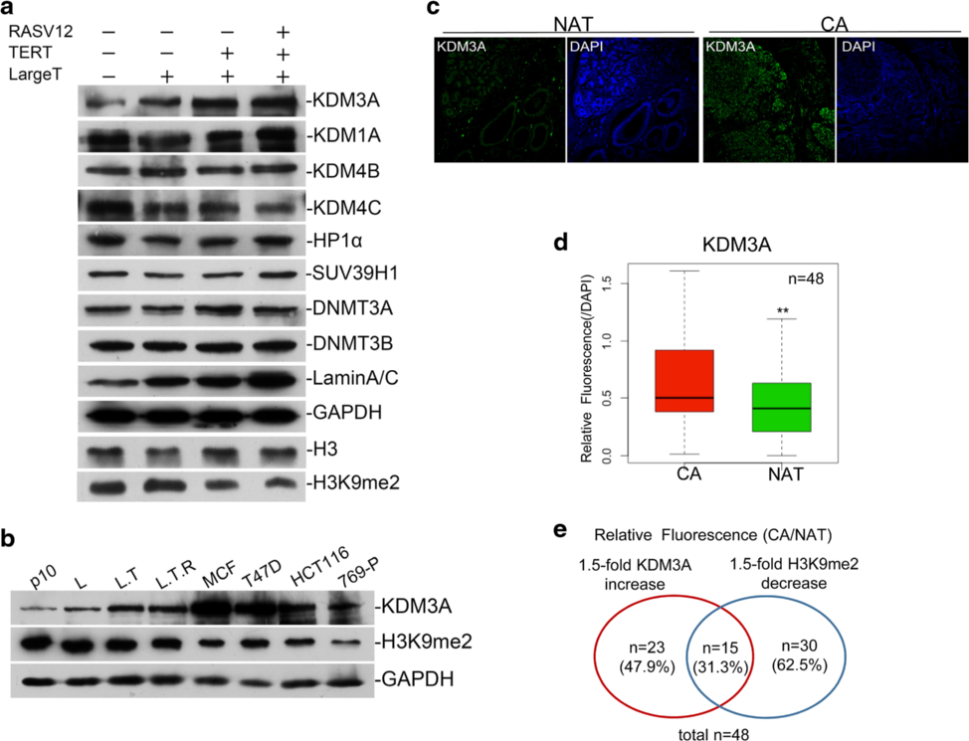
Overexpression of KDM3A/JMJD1A in breast cancer cells and tissues. **a** Analysis of mentioned histone H3K9 methyltransferases and demethylases by Western blotting; KDM3A/JMJD1A levels gradually increase with transformation. **b** High expression of KDM3A/JMJD1A in breast cancer cell lines MCF7 and T47D. **c** Tissue array with paired breast cancer and normal tissues was used for KDM3A staining. **d** The average KDM3A level in breast cancer tissues is significantly higher than that in the paired normal tissues (*n* = 48). **e** Venn diagrams to show that 15 out of 48 paired samples showed both 1.5-fold KDM3A increase and 1.5-fold H3K9me2 decrease in the cancer tissues

After determining the reduction of H3K9 methylation as a frequent event in breast cancer, we started to investigate the underlying molecular mechanisms. We firstly examined the mRNA levels of all the known H3K9 methyltransferases and demethylases by RNA-seq and quantitative RT-PCR, but none matched the observed H3K9 methylation pattern (Additional file 1: Figure S5A, B). We then asked if the regulations of these enzymes take place at the protein level. We surveyed the protein levels in transformed cell line with all the available antibodies and found that KDM3A/JMJD1A, a demethylase for H3K9me1 and me2, gradually increased during transformation, inversely matching the decrease of H3K9me2 (Fig. 4a and Additional file 1: Figure S5C). We further found that KDM3A/JMJD1A is much higher in two breast cancer cell lines, MCF and T47D, than that in the primary HMC and other two cancer cell lines, HCT116 and 769-P (Fig. 4b). A commercial breast tissue array containing 48 pairs was stained with KDM3A and the statistical analysis showed that KDM3A significantly increases in breast cancer tissues compared with normal tissues (Fig. 4c, d). Another piece of array from the same batch was stained with H3K9me2. Fifteen of 48 pairs (31.3 %) showed both KDM3A increase and H3K9me2 decrease. Taken together, these data show that histone H3K9 demethylase, KDM3A/JMJD1A, increases in breast cancer cell lines.

Considering the inconsistency of its mRNA and protein level, KDM3A/JMJD1A is probably regulated at the post-translational level. To further verify it, MG132 (an inhibitor for proteasome) or chloroquine (CQ, an inhibitor for lysosome) was used to treat HMC cell line. Both drugs increased the protein level of KDM3A/JMJD1A (Additional file 1: Figure S5D), suggesting its stability was controlled by both proteasome and lysosome. To verify the function of KDM3A/JMJD1A, we expressed its wild type or catalytic dead mutant (H1180A) and confirmed the expression of wild type decrease H3K9me2 in the cell (Additional file 1: Figure S5E).

### H3K9 dimethylation and transcription of cancer-related genes regulated by KDM3A/JMJD1A

**Fig. 5:**
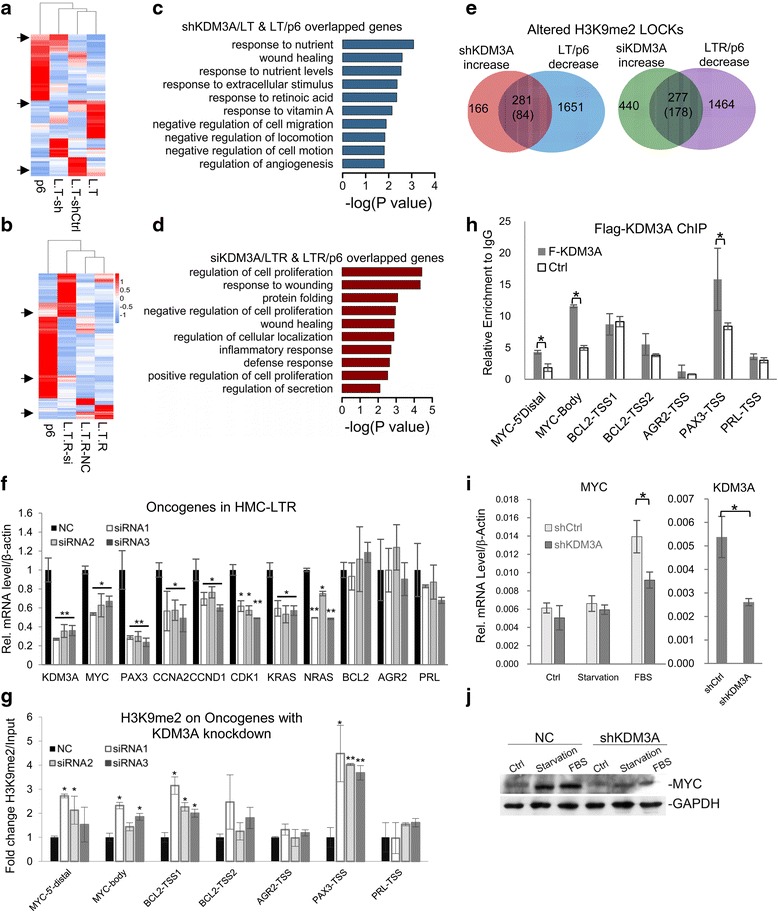
KDM3A/JMJD1A regulates the transcriptional program of multiple oncogenes during transformation. **a**, **b** Expression of DEGs in KDM3A/JMJD1A-deficient cell lines and LT/p6 or LTR/p6 was shown in the heatmap. **c**, **d** Biological process analysis of DEGs which were rescued by KDM3A deficiency in transformed cells back to the level in primary cell (marked by *arrows* in **a** and **b**). **e** Venn diagrams reveal the amount of decreased H3K9me2 LOCKs in LT or LTR restored by KDM3A/JMJD1A knockdown. **f** KDM3A/JMJD1A knockdown reduced the mRNA level of oncogenes in HMC-LTR. **g** KDM3A/JMJD1A deficiency increased H3K9me2 level on *MYC*, *BCL2*, *PAX*, *PRL*, and *AGR2 locus*. **h** A cell line stably expressing F-KDM3A/JMJD1A was established from breast cancer cell line T47D. ChIP assay with anti-Flag was performed as indicated. **i**, **j** Serum was withdrawn from the medium culturing T47D cells for 24 h, and FBS was added back to induce MYC expression. KDM3A deficiency reduced MYC mRNA and protein induction. *P < 0.05, **P < 0.01

To further investigate the role of KDM3A/JMJD1A in regulating transformation, we knocked it down in HMC-LTR using small interfering RNA (siRNA) and found that KDM3A/JMJD1A deficiency rescued the expression of most cancer-related genes in HMC-LTR to the levels in primary cells (Additional file 1: Figure S6A). We performed RNA sequencing and found that KDM3A/JMJD1A deficiency mainly affected the genes involved in cell proliferation, cell cycle, wound healing, and protein transport (Additional file 1: Figure S6B, C). We took the DEGs in HMC-LT (Fig. 5a, Additional file 2: Tables S12 and S13) and HMC-LTR (Fig. 5b, Additional file 2: Tables S14 and S15), respectively, which were rescued by KDM3A/JMJD1A knockdown to the levels in primary cell, and performed GO analysis. The biological processes of these genes are very similar to those changed during transformation (Fig. 5c, d), indicating KDM3A/JMJD1A is the key factor for the process. H3K9me2 ChIP-seq analysis in HMC-LT further showed that the increased LOCKs by KDM3A/JMJD1A knockdown largely overlapped with the decreased LOCKs in transformation (Fig. 5e, left). The analysis in HMC-LTR showed similar results (Fig. 5e, right). KDM3A/JMJD1A deficiency also restored a large portion of the decreased H3K9me2 peaks in HMC-LT and -LTR cells (Additional file 1: Figure S6D). The increased genes with KDM3A knockdown were also studied with GO analysis (Additional file 1: Figure S6E).

When KDM3A/JMJD1A was knocked down in HMC-LTR, the mRNA of most of these genes decreased, suggesting KDM3A may directly regulate their transcription (Fig. 5f). The ChIP-seq results were verified with quantitative PCR and we found that KDM3A/JMJD1A regulates H3K9me2 on a group of oncogenes, including *MYC*, *PAX3*, *AGR2*, *PRL*, and *BCL2* (Fig. 5g). We then performed Flag ChIP analysis in an F-KDM3A/JMJD1A stable cell lines derived from a breast cancer cell line T47D. The results indicated that KDM3A/JMJD1A binds directly on oncogenes *MYC* and *PAX3* in T47D (Fig. 5h). We speculated that additional signals are probably required to activate *MYC* expression. We induced *MYC* in T47D breast cancer cell line with FBS treatment after serum starvation. The elevation of *MYC* mRNA was impaired in the absence of KDM3A (Fig. 5i), as well as its protein (Fig. 5j). All these results demonstrated that KDM3A/JMJD1A regulates breast tumor transformation through directly binding *MYC* and *PAX3* oncogenes and modulating their transcription.

### KDM3A/JMJD1A deficiency impairs the growth and migration of breast cancer cells

**Fig. 6:**
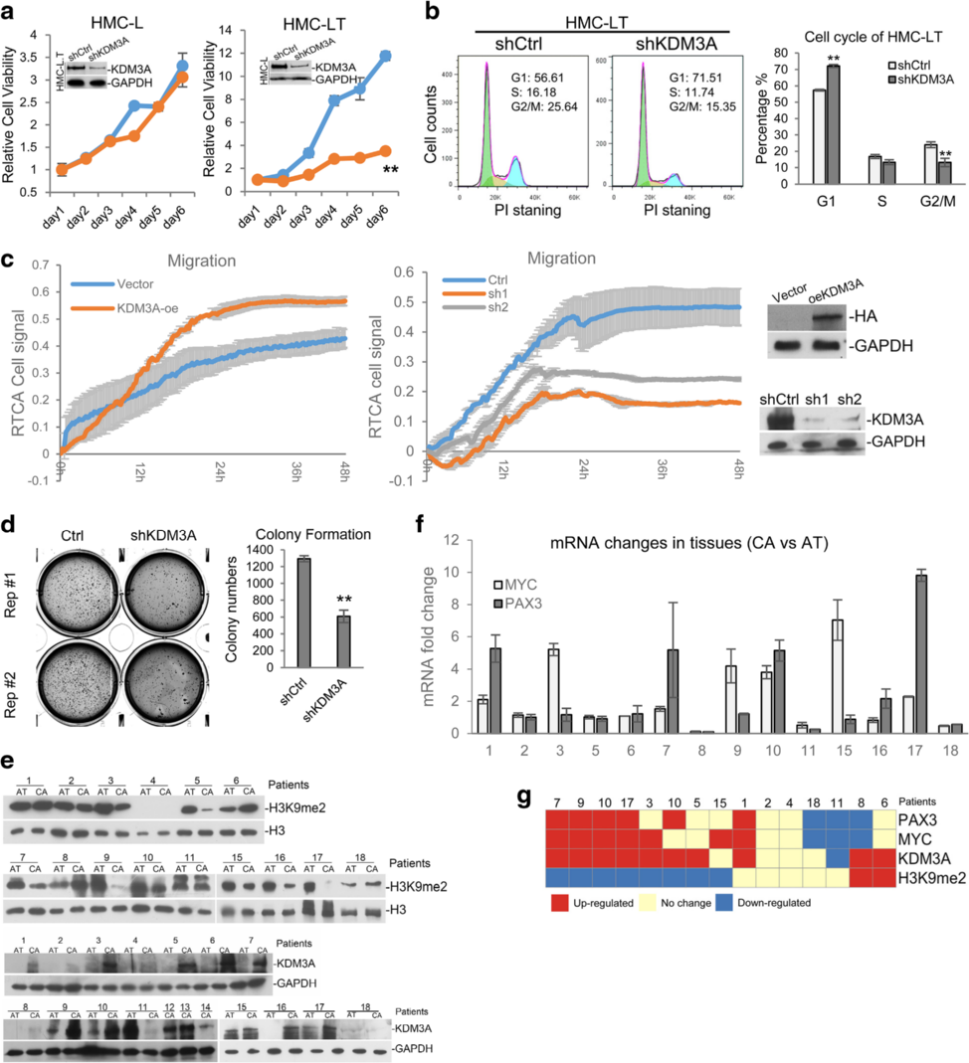
The regulation of tumorigenesis by KDM3A. **a** *KDM3A* stable knockdown cell lines were made in HMC-L and -LT. Cell viability was measured by MTT assay. **b** KDM3A/JMJD1A was knocked down in HMC-LT, and cell cycle was analyzed by flow cytometry with PI staining. **c** RTCA cell migration assay was performed as described with KDM3A/JMJD1A overexpression and knockdown cells. **d** KDM3A/JMJD1A was stably knocked down in T47D breast cancer cell line. Cells were seeded in soft agar and colonies were counted 4 weeks later. Experiments were repeated three times, with two duplicates in each experiment. **e** The protein level of KDM3A and H3K9me2 in the collected breast cancer and paired adjacent tissues is measured with western. **f** The mRNA levels of *MYC* and *PAX3* in the 15 paired cancer and adjacent tissues are measured by quantitative RT-PCR. **g** The level of KDM3A protein, H3K9me2, MYC, and PAX3 mRNA was summarized. *P < 0.05, **P < 0.01

To further explore the function of KDM3A/JMJD1A in breast cancer transformation, we made *KDM3A* stable knockdown cell lines in HMC-L and HMC-LT. We could not get the cell line in HMC-LTR because it was extremely difficult to have four different constructs integrated into one cell line. The results of MTT assay indicated that the growth of HMC-LT was greatly impaired in the absence of KDM3A (Fig. 6a). The cell cycle analysis also indicated that HMC-LT was arrested at G1 phase with KDM3A deficiency (Fig. 6b). The abilities of cell migration and invasion were measured by RTCA real-time monitor or traditional transwell assay with T47D cell line. The results indicated that KDM3A/JMJD1A overexpression enhanced T47D migration and knockdown repressed it (Fig. 6c and Additional file 1: Figure S7A). But cell invasion was not affected (Add Fig. S7B). Colony formation assay indicated that KDM3A/JMJD1A knockdown significantly decreased the colony number in the plate (Fig. 6d). To further test whether MYC and PAX3A really regulate the tumorigenicity ability of the transformed cell, we knock down the two genes respectively in HMC-LTR. The assays performed by RTCA system indicated that MYC and PAX3 are both involved in cell migration and invasion (Additional file 1: Figure S7C). The above data indicated that KDM3A positively regulates the survival and migration of breast cancer cells.

### High expression of KDM3A/JMJD1A in breast cancer tissues

We then asked whether KDM3A/JMJD1A elevation also occurs in breast cancer patients. We analyzed the breast cancer tissues from 18 patients by Western blotting, out of which 15 has the corresponding adjacent tissues. In the 15 paired tissues, 8 pairs showed H3K9me2 reduction in cancer, 10 with KDN3A/JMJD1A increase; 7 with MYC mRNA increase, and 6 with PAX3 mRNA increase; 4 with all the above characteristics (Fig. 6e–g). The results suggested KDM3A/JMJD1A regulates the tumorigenesis of breast cancer through down-regulating H3K9me2 on oncogenes MYC and PAX3.

## Discussion

Development of novel markers and drug targets are keys for precision diagnosis and treatment. In this study, we combine systematic and biochemistry approaches and prove that H3K9me2/3 decreases and KDM3A/JMJD1A increases in tumor cell transformation model and in vivo clinical samples. Epigenomic analysis identifies the dynamic changes of H3K9me2 reduction on *MYC*, *PAX3*, *WNT5A*, and *CDKN2A/B*, are critical events during transformation. These events can be further developed as diagnosis markers and drug targets in clinical research.

Our data suggest that H3K9me2 regulates transformation mainly through transcription. We discovered that the boundaries of decreased H3K9me2 LOCKs are enriched with cancer-related genes. H3K9me2 reduction usually starts from the LOCK boundaries and the boundary genes are relatively easier to be de-repressed and, therefore, can help the cell adapt to different development or transformation cues. The genes in the center regions are difficult to be accessed. This explains how H3K9me2 regulates cell identity in tumor transformation, as well as other processes. In our study, we also found that H3K9me2 reduction may not be always related with increased transcription of some genes. For example, H3K9me2 on MYC is gradually reduced during transformation, but its mRNA level keeps constant. However, we found that its induced transcription is regulated by KDM3A/JMJD1A transient knockdown with siRNA. These data further indicated that changes of histone modifications may not affect transcription immediately, but it will be critical during some special circumstances. This has also been observed with other modifications [40].

KDM3A/JMJD1A is a gene involved in sex determination [41, 42]. While we are submitting the manuscript, one group reported that KDM3A/JMJD1A regulates the expression of ER target genes in breast cancer cells [43], which supports our discovery of KDM3A/JMJD1A’s role in breast cancer. Beyond this, we provided genome-wide H3K9me2 map in the absence of KDM3A/JMJD1A and identified *MYC* and *PAX3* as direct target genes of KDM3A/JMJD1A. *MYC* is a well-known oncogene for breast cancer, as well as many other cancer types. Mutations in *PAX3* are associated with Waardenburg syndrome, craniofacial-deafness-hand syndrome, and alveolar rhabdomyosarcoma. Above all, our discovery provides new mechanisms for tumorigenesis regulated by KDM3A/JMJD1A.

Taken together, our study reveals the dynamic changes occurring at the boundary regions of H3K9me2 LOCKs, identifies key epigenetic events on cancer-related genes, and proposes their crucial roles in dynamically regulating cell transformation. The work not only provides potential diagnosis markers and drug targets for future clinical research but also puts forward novel concepts for epigenomic studies.

## Conclusions

Our study demonstrates that the levels of H3K9me2 and me3 decrease during breast cancer cell transformation in vitro and in patient tissues. ChIP analysis revealed that the genes localized at the boundaries of H3K9me2 LOCKs are related with cancer. The increase of KDM3A, a histone demethylase, is responsible for the reduction of H3K9me2. KDM3A regulates transcription of oncogenes, such as MYC and PAX3, via directly binding to the genes and regulates their H3K9me2 level.

## Methods

### Cell lines and reagents

Human primary mammary cells were purchased from Chi Scientific and cultured in DMEM/F12 (10 % FBS, with addition of insulin, hydrocortisone, and EGF) at 37 °C with 5 % CO_2_. MCF7 was purchased from Cell Bank of Chinese Academy and cultured in DMEM with FBS, insulin (10 μg/mL), sodium pyruvate (Invitrogen), and nonessential amino acid (Invitrogen); T47D is a gift from Dr. Yong-Feng Shang of Tianjin Medical University and cultured in RPMI1640 with FBS and insulin. Both the primary cells and cancer cell lines were sub-cultured 1:4 on reaching confluence; each passage was considered two PD.

Antibodies were purchased from the indicated companies: H3K9ac, hTERT, KRT14, and ACTG (Epitomics); KRT18 (ProteinTech), JMJD1B, JMJD2B, JMJD2A, H4K20me3, H3K79me2, E-cadherin, and RAS (CST); H3K4me1, H3K4me3, H3K27me3, H3K27me1, H3K27me2, and H3K36me2 (Millipore); H3, H3K9me1, H3K4me2, and H3K36me3 (Abcam); H3K9me2, H3K9me3, GAPDH, EHMT2, SUV39H1, CBX5, DNMT3A, DNMT3B, DNMT1, and KDM1A (Abclonal); KDM3A/JMJD1A (Abclonal for western and Millipore for immunostaining). The information of primers and siRNAs are listed in Additional file 2: Table S16.

### Transformation of human breast tumor cell

Transformed breast cell lines were generated as previous described. 293FRT cells were co-transfected with packaging plasmid ZV77 (psPAX and pMD2G for lentivirus) and pBabe retroviral plasmids containing desired complementary DNA (cDNA). Supernatants containing virus were harvested 48 h later and HMC was infected together with 8 μg/ml polybrene. Typically, more than 80 % of cells were infected as measured by parallel infections with a GFP-expressing construct. Drug selection was performed with 200 μg/ml G418 for neomycin, 50 μg/ml hygromycin, or 0.5 μg/ml puromycin. pBabe with large T antigen, hTERT, or RAS (V12) were purchased from Addgene.

### Immunofluorescent staining of cancer tissues and cultured cells

Tumors tissue array (Alenabio, www.alenabio.com) were fixed in 10 % formalin, embedded in paraffin, and followed by standard dewaxing procedures. Cells were cultured on the cover slips and fixed with freezing methanol after washing twice in PBS. The cover slips or tumors tissue array were then washed three times by PBS and blocked in PBS with 1 % BSA for 10 min or 1 h. The cover slips or tumor tissue arrays were hybridized with first and second antibodies for 1 h, respectively. Then, the slips were mounted with prolong anti-fade kit (Invitrogen) and observed with fluorescent microscopy. The arrays used for staining are as follows: H3K9me2 - BR243K, BR243L, BR243M, BR724, BR725, BCN963a; H3K9me3 - BR243B, BR243D, BR243K, BR243L, BR243A, BC081120, BCN963a; and KDM3A - BR724, BR725.

### Reverse transcription and quantitative PCR

Cells were scraped down and collected by centrifugation. Total RNA was extracted with RNA extraction kit (Yuanpinghao) according to manufacturer’s manual. Approximately 1 μg of total RNA was used for reverse transcription with a first-strand cDNA synthesis kit (Toyobo). The amount of mRNA was assayed by quantitative PCR. β-Actin was used to normalize the amount of each sample. Assays were repeated at least three times. Data shown were average values ± SD of one representative experiment. All primer sequences are presented in Additional file 2: Table S16.

### ChIP assay

ChIP assay was performed as previously described [40]. Briefly, approximately 1 × 10^7^ cells were fixed with 1 % formaldehyde and quenched by glycine. The cells were washed three times with PBS and then harvested in ChIP lysis buffer (50 mM Tris-HCl, pH 7.6, 1 mM CaCl_2_, 0.2 % Triton X-100). DNA was digested to 150–300 bp by MNase (Sigma) before extensive centrifugation. Four volumes of ChIP dilution buffer (20 mM Tris-HCl, pH 8.0, 150 mM NaCl, 2 mM EDTA, 1 % Triton X-100, 0.1 % SDS) was added to the supernatant. The resulted lysate was then incubated with protein G beads and antibodies at 4 °C over night. The beads were washed five times and DNA was eluted by Chip elution buffer (0.1 M NaHCO_3_, 1 % SDS, 20 μg/ml proteinase K). The elution was incubated at 65 °C over night and DNA was extracted with DNA purification kit (TIANGEN). The purified DNA was assayed by quantitative PCR with Biorad MyIQ. Assays were repeated at least three times. Data shown were average values ± SD of representative experiments. The sequences of primers are in Additional file 2: Table S16.

### Pipeline of RNA-seq analysis

mRNA-seq library was performed by using Illumina TruSeq library construction kit. A 5 μg of total RNA was used as initiation and then prepared according to the manufacturer’s instruction. mRNA-seq libraries were sequenced using HiSeq2000 for 100-bp paired-end sequencing. Quality control of mRNA-seq data was performed using Fatsqc, and then low quality bases were trimmed. After quality control, data were mapped to hg19 genome reference by Tophat2 and allow maximum 2 mismatch. Cufflinks were used to find out differential expression genes. Gene ontology analysis was performed using DAVID (http://david.abcc.ncifcrf.gov) [44, 45].

### Pipeline of ChIP-seq analysis

ChIP was performed using desired antibodies. Library was prepared using Illumina TruSeq kit according to the manufacturer’s procedure. Briefly, DNA was prepared for end repair and “A” tailing, adaptor ligation, and library amplification. ChIP-seq libraries were sequenced on HiSeq 2000 for 100-bp paired-end sequencing.

Quality control of ChIP-seq data was performed using Fastqc, and then low quality bases and adaptor contamination were deleted. After quality control and data filtering, data were mapped to hg19 using BWA aln algorithm. Since H3K9me2 and H3K9me3 appear large scale in chromatin, SICER software was used for peaks calling with window size 1000 and gap size 10,000. H3K9me2 and H3K9me3 enrichment region gene annotation was performed using RefSeq gene reference [46]. Gene ontology analysis and KEGG pathway analysis were performed using DAVID.

### TCGA breast cancer differential expression gene analysis

The gene expression data pf 100 paired breast cancer and normal tissues were downloaded from TCGA data portal for analysis of differential expressed genes (https://tcga-data.nci.nih.gov/tcga/, the data of total 101 pairs were downloaded but that of one cancer tissue were not readable, so only 100 pairs were used). In order to figure out significant differential expression genes between cancer sample and normal tissue, genes’ expression level less than 5 FPKM in all 201 samples were deleted, and then ANOVA analysis was performed for the rest genes with *P* value cutoff 0.001. After ANOVA analysis, genes’ average expression level between cancer and normal tissues less than twofold change was deleted. Gene ontology analysis of differential expression genes was performed using DAVID.

### Cell cycle analysis with flow cytometry

Cells were harvested after digestion with 0.05 % Trypsin-EDTA. The cells were then washed twice with PBS and fixed in ice-cold 70 % ethanol overnight. Fixed cells were washed twice with PBS and stained in PBS containing propidium iodide (PI, 50 μg/mL) and RNase (100 μg/mL) for 30 min at 37 °C. Cell cycle analysis was performed on an Epics XL-MCL flow cytometer (Beckman Coulter) with System II (version 3.0) software (Beckman Coulter). Additional analysis of cell cycle distribution was determined using Flowjo software.

### Cell viability assay

Cell viability was performed by the MTT assay as previously described [47]. Briefly, cells were split at 1 × 10^3^ per well in 96-well plates. Next every 24 h, the cells were added with MTT (0.25 μg) in each well for 4 h at 37 °C; the medium with the formazan sediment was dissolved in 50 % DMF and 30 % SDS (pH 4.7). The absorption was read at 570 nm.

### Colony formation assay

The bottom layer of 0.6 % agar noble in medium was first placed onto 6-well plate. Cells were seeded in 0.35 % top agar containing medium. Fresh top agar was added 1.5 weeks later, and colonies were counted 8 weeks later. For HMCs, the 50,000 cells were seeded while 5000 or 10,000 cells were seeded for T47D.

### Cell migration and invasion assay

The RTCA assay was done as the manufacturer’s protocol. Cells were cultured at 6000 per well in CIM-Plate wells coated with (invasion) or without (migration) matrigel. The cell index signals were read by xCELLigence RTCA DP Analyzer (ACEA Bioscience Inc.). Invasion and migration are monitored continuously over a 48-h period.

The transwell assay was done as follows. Briefly, cells were split at 1 × 10^5^ per well in 24-well transwell plates coated with (invasion) or without (migration) matrigel. The cells were fixed in 4 % PFA and stained by crystal violet after 48 h. The positive cells were counted under microscope.

Each experiment was repeated three times and results were presented as mean ± SD.

### Cancer tissue collection

All the cancer tissues are collected after obtaining the consents of the patients. All the experiments are carried out in accordance with the approved guidelines and protocols by Medical Ethics Committee of Zhongnan Hospital, Wuhan University.

### Data access

The data have been uploaded to GEO database and can be found at the following URL: http://www.ncbi.nlm.nih.gov/geo/query/acc.cgi?acc=GSE64367.

## Additional files

Additional file 1: Figures S1–S7.
**Figure S1.** Establishment of four-stage breast cancer model. **Figure S2.** Gene expression profiling of TCGA breast cancer tissues and transformed HMC cell lines. **Figure S3.** Analysis of H3K9me2 peaks on chromatin. **Figure S4.** Transcriptional program change of genes with decreased H3K9me2. **Figure S5.** RNA and protein levels of epigenetic enzymes in the tumor model cell lines. **Figure S6.** KDM3A deficiency impairs the transcriptional program of cancer-related genes. **Figure S7.** KDM3A regulates the growth of breast cancer cells. (PDF 4169 kb) Additional file 2: Tables S1–S16.
**Table S1.** Differential expression genes between L and P6 cell lines. **Table S2.** Differential expression genes between LT and P6 cell lines. **Table S3.** Differential expression genes between LTR and P6 cell lines. **Table S4.** DEGs between LT and L cell lines. **Table S5.** DEGs between LTR and LT cell lines. **Table S6.** DEGs between TCGA breast cancer and normal tissues (≥threefolds). **Table S7.** Genes overlapped between DEGs of TCGA and transformed cell lines. **Table S8.** Genes localized in H3K9me3 small LOCKs of HMC-p6. **Table S9.** Genes localized in decreased H3K9me2 boundaries in HMC-LT. **Table S10.** Genes localized in decreased H3K9me2 boundaries in HMC-LTR. **Table S11.** Uniprot cancer-related genes in the boundaries of decreased H3K9me2 LOCKs (compared to HMC-p6). **Table S12.** Genes overlapped between LT_NC/LT_shKDM3A and LT/P6 DEGs. **Table S13.** DEGs between LT_NC and LT_shKDM3a cells. **Table S14.** Genes overlapped between LTR_NC/LTR_siKDM3A and LTR/P6 DEGs. **Table S15.** DEGs between LTR_NC and LTR_siKDM3a cells. **Table S16.** Primer and siRNA sequences. (XLS 1135 kb)
