# Supplementary material for: Global histone modification profiling reveals the epigenomic dynamics during malignant transformation in a four-stage breast cancer model
Source: Clin Epigenetics. 2016 Mar 31;8:34. doi: 10.1186/s13148-016-0201-x (PMC4815258; doi:10.1186/s13148-016-0201-x)
Supplement: Additional file 1: Figures S1–S7. — Figure S1. Establishment of four-stage breast cancer model. Figure S2. Gene expression profiling of TCGA breast cancer tissues and transformed HMC cell lines. Figure S3. Analysis of H3K9me2 peaks on chromatin. Figure S4. Transcriptional program change of genes with decreased H3K9me2. Figure S5. RNA and protein levels of epigenetic enzymes in the tumor model cell lines. Figure S6. KDM3A deficiency impairs the transcriptional program of cancer-related genes. Figure S7. KDM3A regulates the growth of breast cancer cells. (PDF 4169 kb) [file 13148_2016_201_MOESM1_ESM.pdf]

Supplementary Figure 1

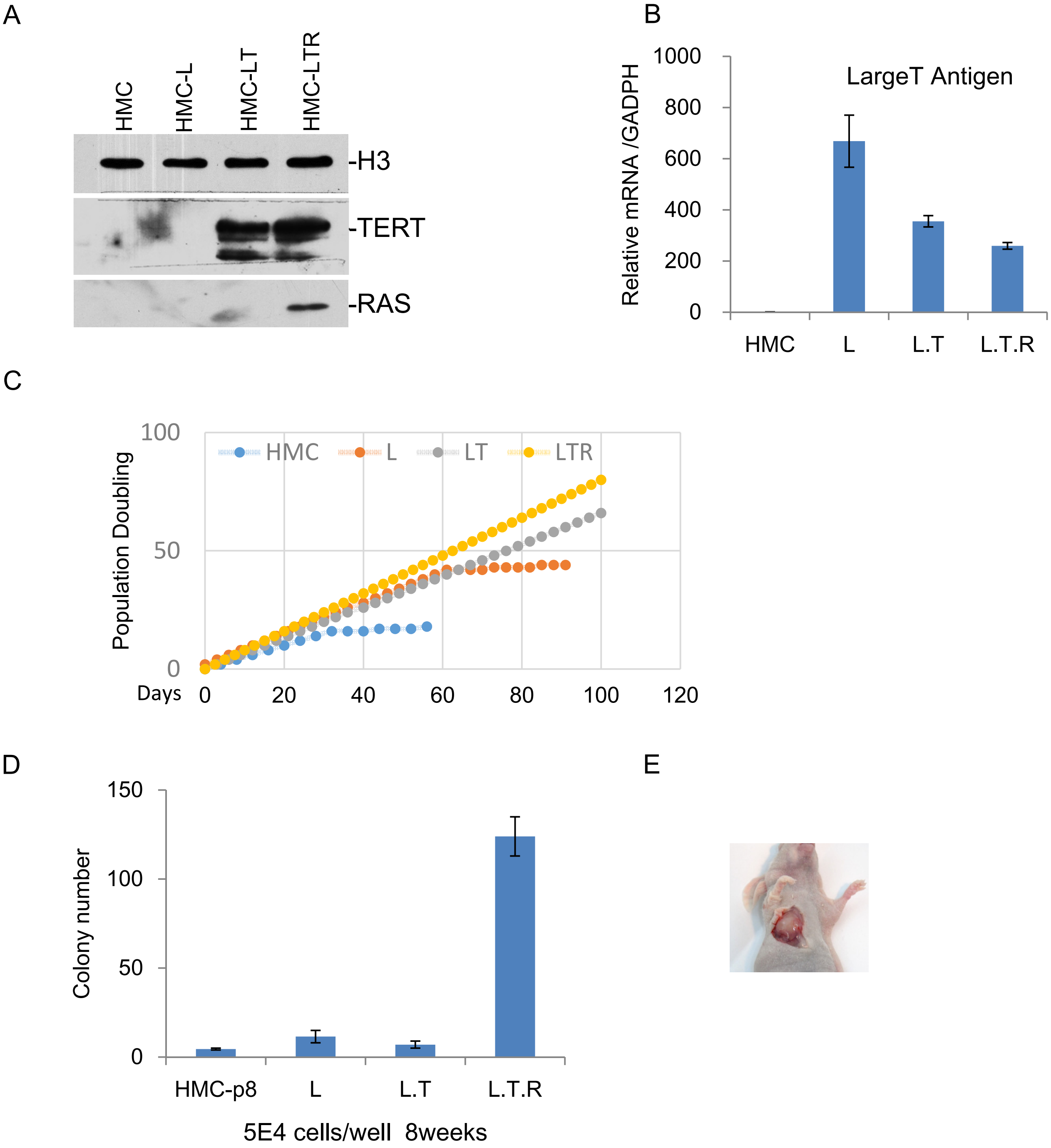

Supplementary Figure 2

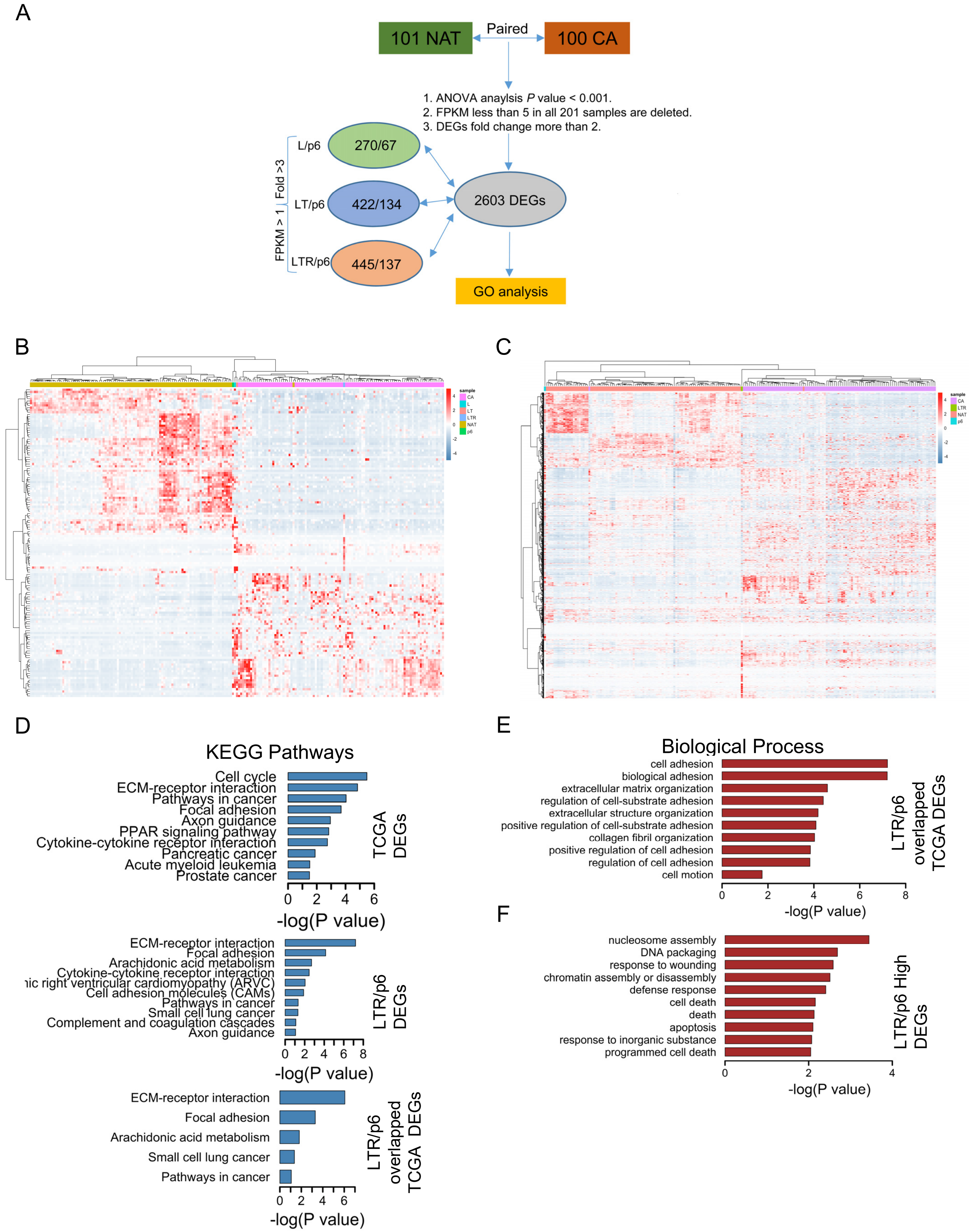

Supplementary Figure 2

G

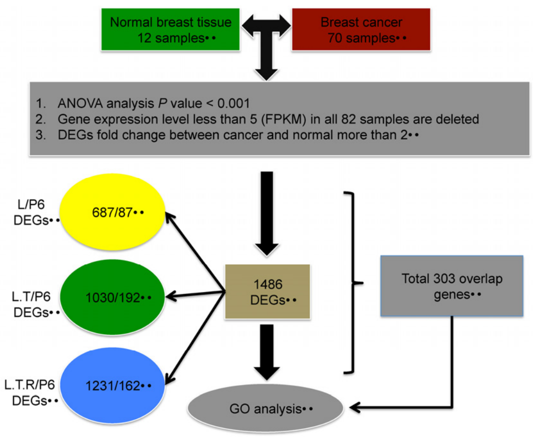

H

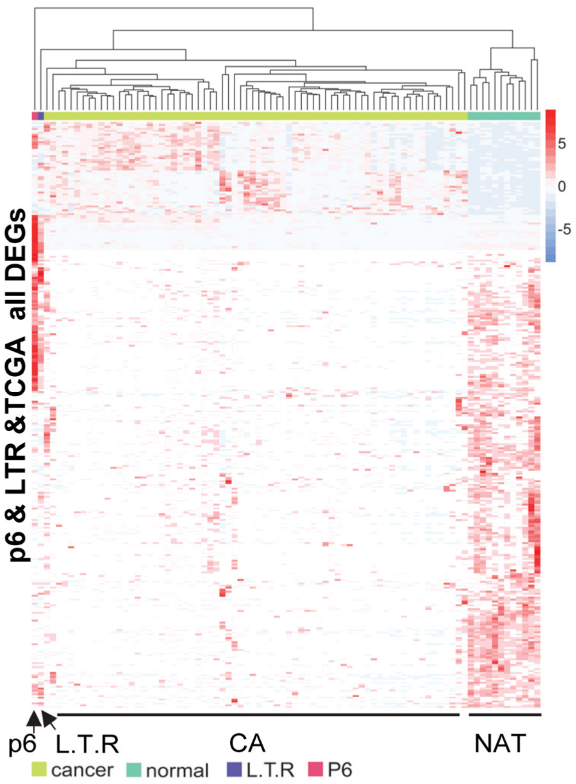

Supplementary Figure 3

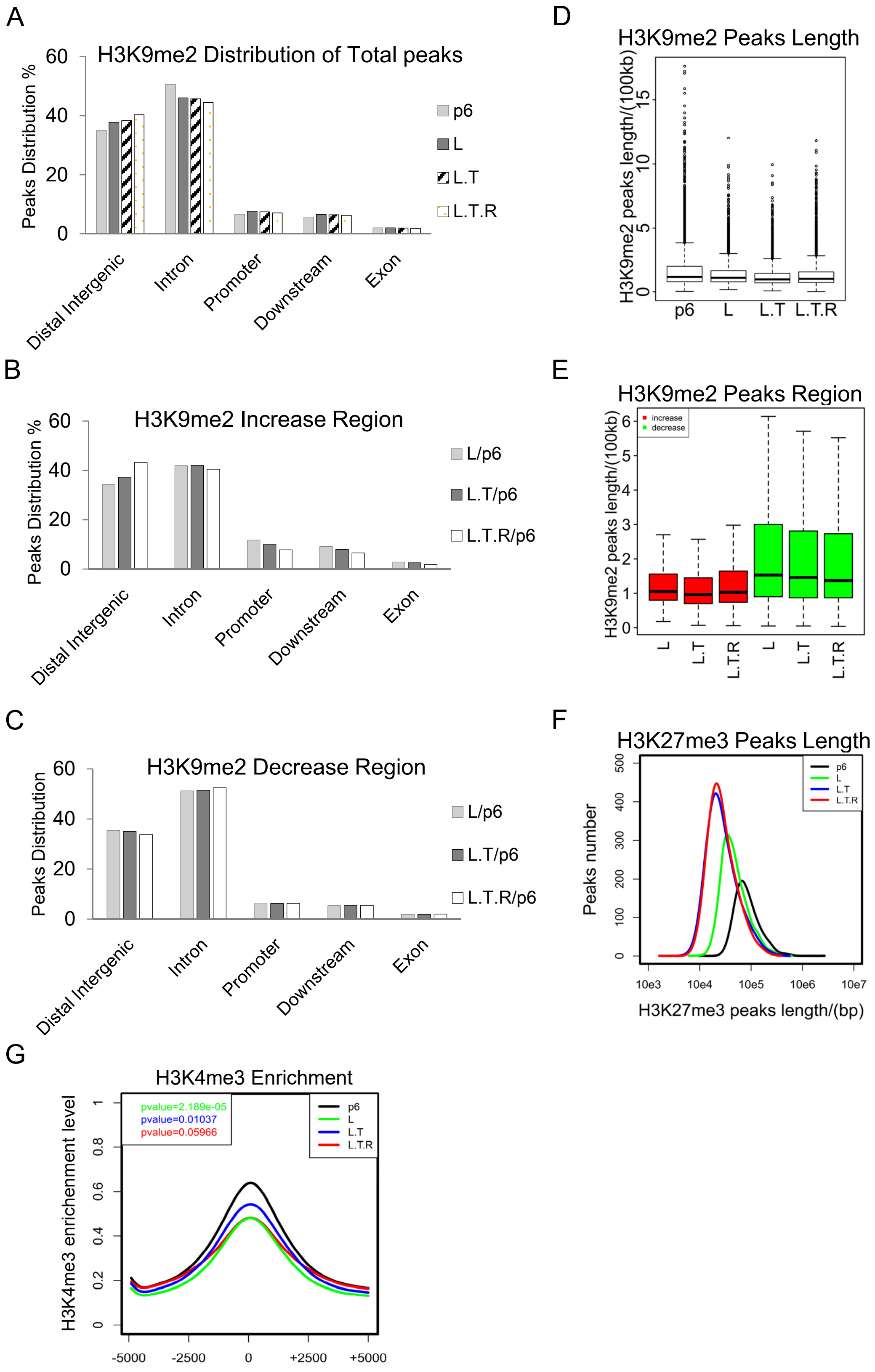

Supplementary Figure 4

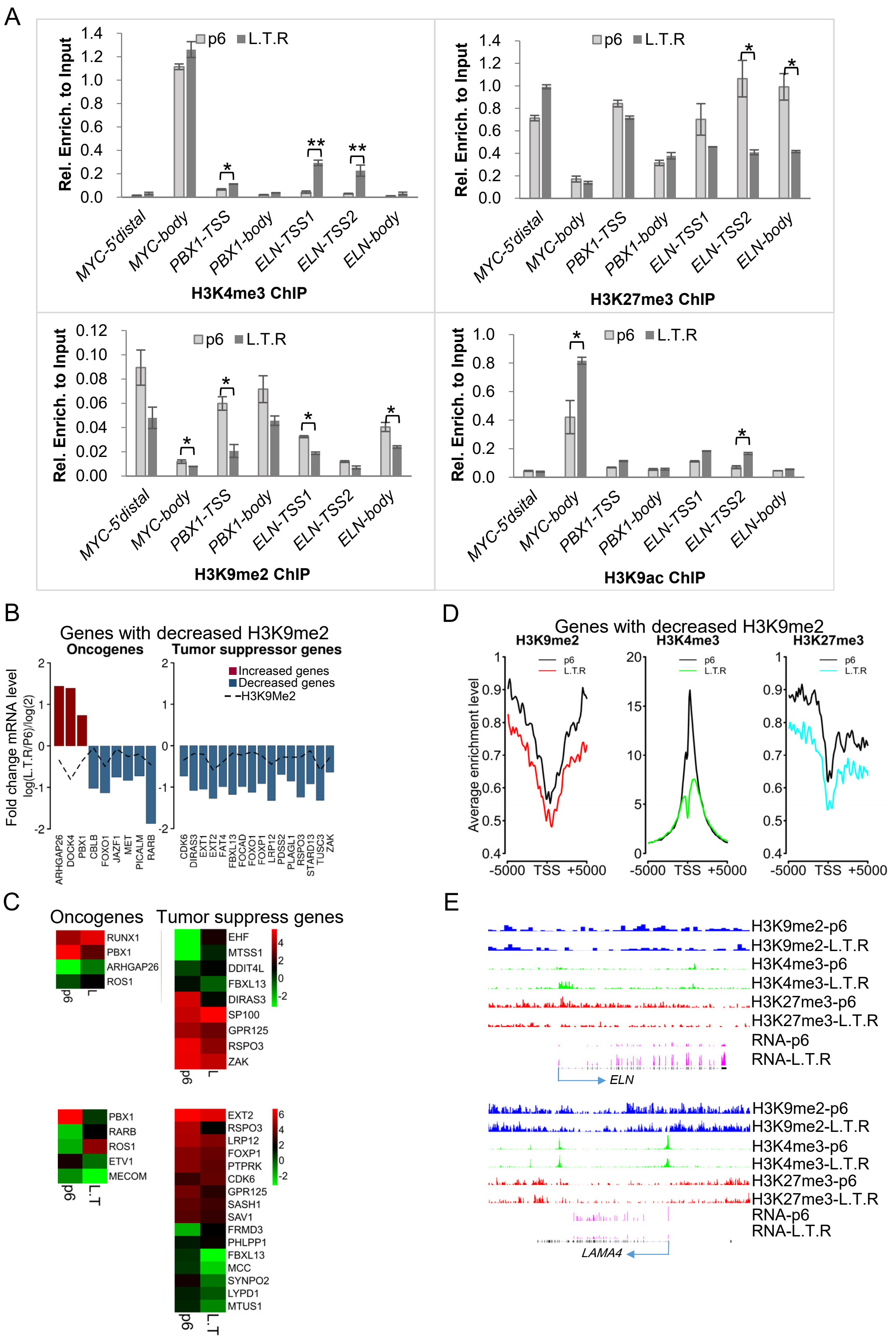

Supplementary Figure 5

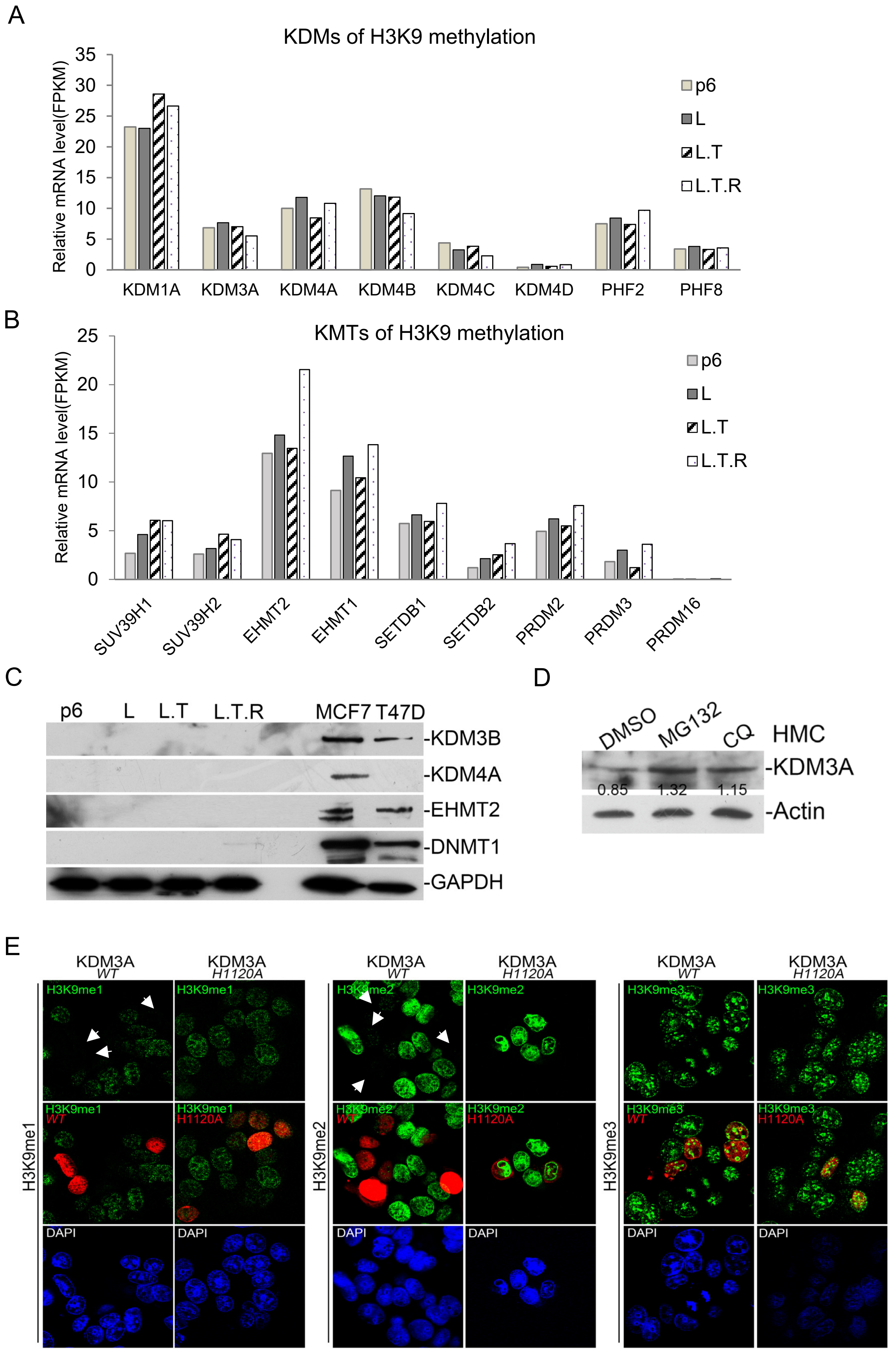

Supplementary Figure 6

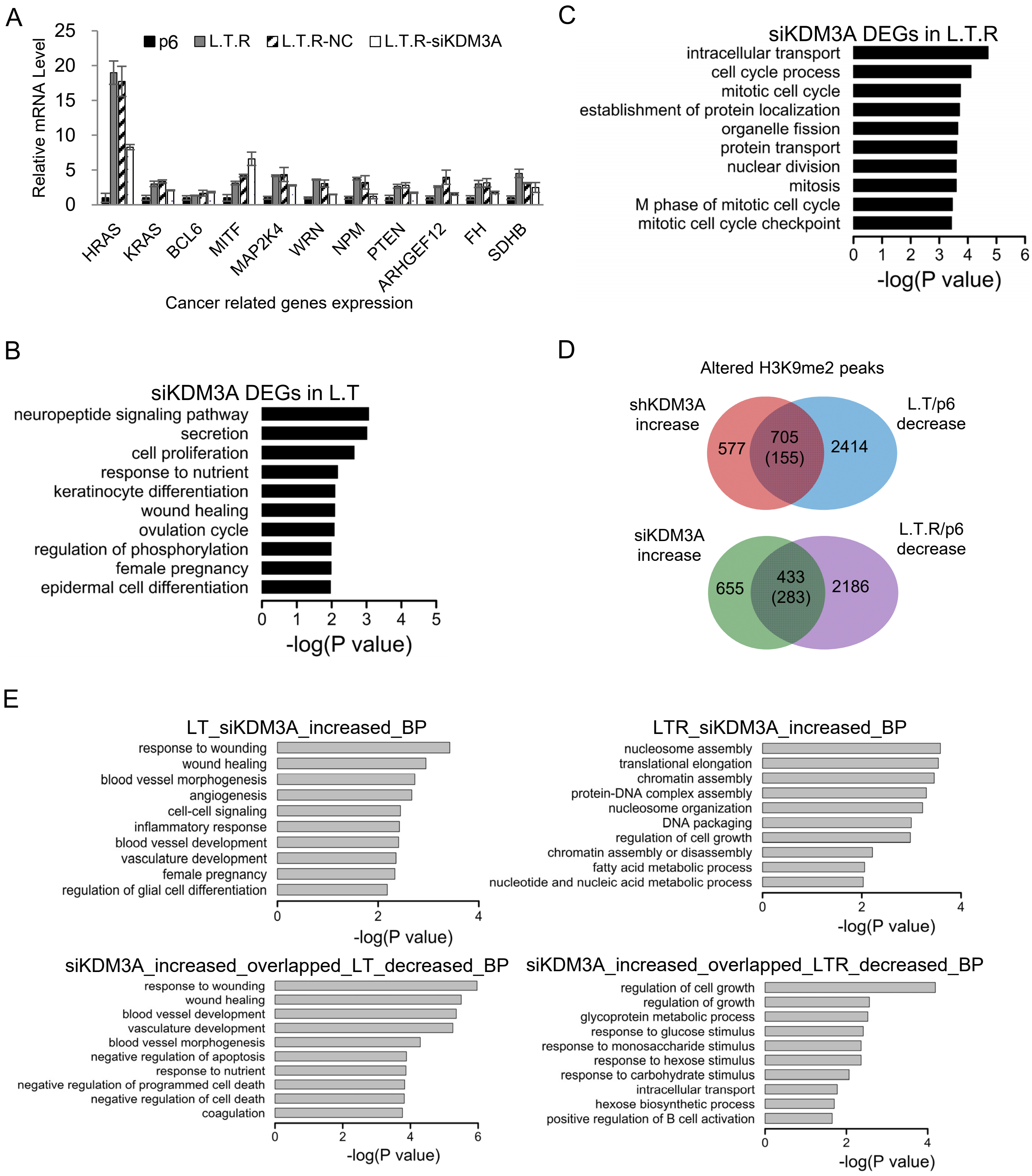

Supplementary Figure 7

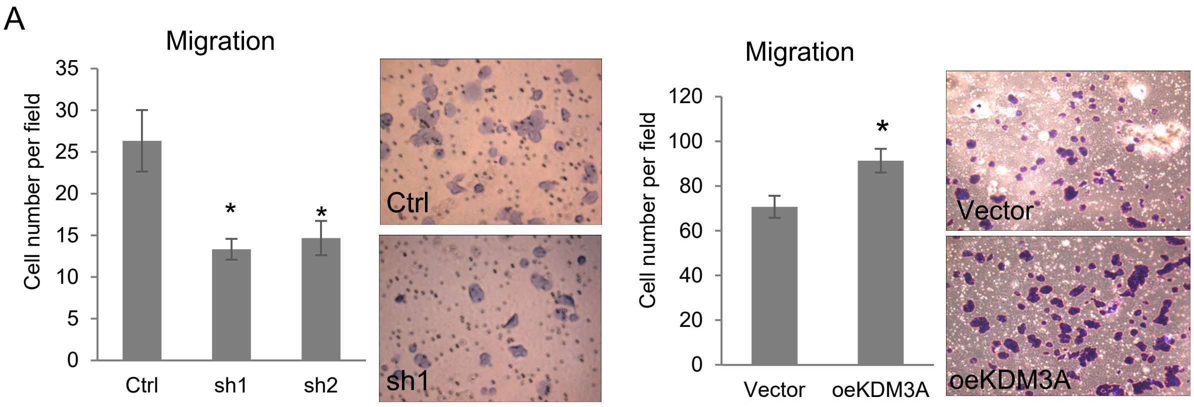

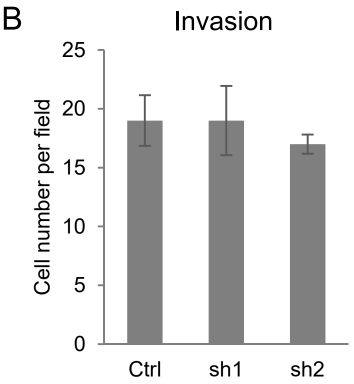

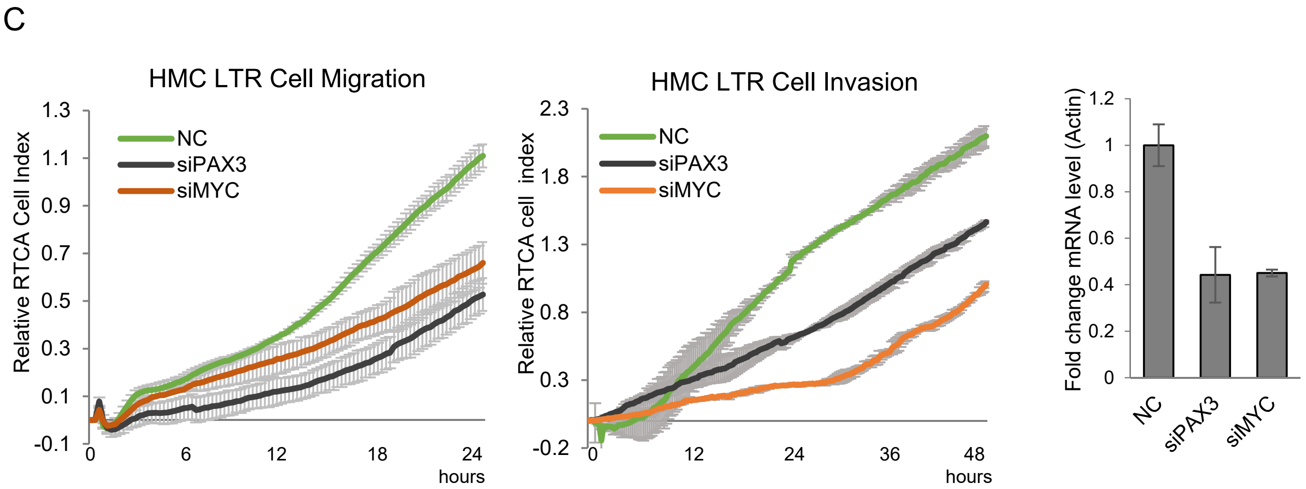

Supplementary figure legends

Fig. S1 Establishment of four-stage breast cancer model. (A) Three genes were stably expressed in human primary mammary cell respectively *via* retroviral infection. Four cell lines were generated for following studies, namely HMC-p6 (human primary mammary cell, passage 6), HMC-L (HMC with large T stable expression), HMC-LT (HMC with large T and *TERT* stable expression) and HMC-LTR (HMC with large T, *TERT* and *HRAS (V12)* stable expression). The expression of TERT and RAS were verified by western. **(B)** Expression of Large T antigen in the four cell lines was verified with RT-PCR. **(C)** The ability of population doubling of the four cell lines were recorded during tissue culture. **(D)** The ability to form colonies of the four cell lines was studied in soft agar. **(E)** The same amount cells of the four cell lines were injected into nude mice and only HMC-LTR was capable to grow into tumor.

Fig. S2 Gene expression profiling of TCGA breast cancer tissues and transformed HMC cell lines. (A) Pipeline of DEG analysis between TCGA 100 paired breast cancer tissues and transformed breast cancer cell lines. **(B)** Clustering of HMC-p6, -L, -LT and -LTR with TCGA samples, using genes analyzed from (A). **(C)** The DEGs of HMC-p6 and –LTR were used for clustering, which nicely distinguished TCGA breast cancer and paired natural tissues. **(D)** KEGG pathways enrichment analysis of TCGA, LTR/p6, LTR/p6 and TCGA overlapped DEGs. **(E)** BP enrichment analysis of TCGA overlapped DEGs with LTR/p6. **(F)** BP enrichment analysis of up regulated genes among LTR/p6 DEGs. **(G)** The pipeline of an early analysis with random TCGA tissues and transformed cell lines. **(H)** The result of an early analysis with random TCGA tissues.

Fig. S3 Analysis of H3K9me2 peaks on chromatin. (A) Chromatin distribution of H3K9me2 in the four cell lines. **(B & C)** Chromatin distribution of increased (B) and decreased (C) H3K9me2 regions. **(D)** Average length of H3K9me2 in the four cell lines in comparison with HMC-p6. **(E)** Average peak length of increased and decreased H3K9me2 peaks in the transformed cell lines. **(F)** Peak numbers of different sized H3K9me2 peaks in the four cell lines. **(G)** The enrichment analysis of H3K4me3 ChIP-Seq data.

Fig. S4 Transcriptional program change of genes with decreased H3K9me2. (A) H3K4me3, H3K9me2, H3K27me3, and H3K9ac in *MYC, PBX1, ELN* gene locus measured by ChIP-qPCR. Primers are designed according to ChIP-seq data. $^*P < 0.05$, $^{**}P < 0.01$. **(B)** Expression changes of oncogenes and tumor suppressor genes at H3K9me2 decreased regions in HMC-p6 and LTR cell lines. **(C)** Transcriptional changes of oncogenes and tumor suppressor genes at H3K9me2 decreased regions in indicated cell lines. Hierarchical cluster heatmap were produced using Euclidean distance and complete cluster method. **(D)** Decrease in H3K4me3 and H3K27me3 on genes at H3K9me2 decreased regions. **(E)** H3K4me3, H3K27me3 and mRNA levels in HMC-p6 and LTR of two gene examples with decreased H3K9me3.

Fig. S5 RNA and protein levels of epigenetic enzymes in the tumor model cell lines. (A, B) mRNA level of KDMs and KMTs of tumor model cell lines are measured with RNA-Seq. **(C)** Western blotting of EHMT2, KDM3B, KDM4A and DNMT1 in HMC tumor model cell lines. **(D)** 10μM MG132 or 20μM Chloroquine (CQ) was used to treat the indicated cell and KDM3A was blotted with its specific antibody. Its relative level *vs* β-actin was quantitated with ImageJ. **(E)** Flag-tagged KDM3A wild type or catalytic dead mutant H1120A was expressed in T47D. H3K9me1, me2, me3 and KDM3A was immunostained repectively.

Fig. S6 KDM3A deficiency impairs the transcriptional program of cancer-related genes. (A) Cancer-related genes were down-regulated by KDM3A knockdown. mRNA level were measured by RT-qPCR. **(B & C)** BP enrichment analysis of KDM3A knockdown in HMC-LT (B) or HMC-LTR (C). **(D)** Venn diagrams reveal decreased H3K9me2 total peaks in HMC-LT or HMC-LTR restored by KDM3A knockdown. Restored peaks are indicated with numbers. $^*P < 0.05$. **(E)** KDM3A was knocked down in HMC-LT or -LTR cells. The indicated DEGs were used for GO analysis.

Fig. S7 KDM3A regulates the growth of breast cancer cells. (A & B) The migration and invasion of KDM3A over expression or knockdown T47D cells were measured with transwell assay. Cells were counted and photographed. **(C)** MYC or PAX3 was knocked down in HMC-LTR cell lines. The migration and invasion ability was measured by RTCA system according to the manufacturer's protocols.
